# Structural features of outdoor latrines influence the abundance of *Anopheles gambiae* s.l. and *Culex quinquefasciatus* in a village in Kisumu County, western Kenya

**DOI:** 10.1186/s13071-025-07011-7

**Published:** 2025-08-27

**Authors:** Noriko Tamari, Heidi E. Brown, Luigi Sedda, Michael A. Riehle, Katherine D. Ellingson, Kathleen R. Walker, Gary L. Christopherson, Harrysone Atieli, Stephen Munga, Kacey C. Ernst

**Affiliations:** 1 Department of Epidemiology and Biostatistics, College of Public Health, University of Arizona, Tucson, AZ USA; 2 Lancaster Ecology and Epidemiology Group, Lancaster Medical School, Lancaster University, Lancaster, UK; 3 Department of Entomology, College of Agriculture and Life Sciences, University of Arizona, Tucson, AZ USA; 4 School of Geography and Development, University of Arizona, Tucson, AZ USA; 5 Sub-Saharan International Center of Excellence for Malaria Research, Tom Mboya University, Homa Bay, Kenya; 6 Kenya Medical Research Institute (KEMRI), Centre for Global Health and Research, Kisumu, Kenya

**Keywords:** *Anopheles*, *Culex*, *Mansonia*, Latrine, Malaria, Abundance

## Abstract

**Background:**

Malaria remains a leading cause of mortality in Kenya, despite concerted efforts in malaria vector control. Reducing outdoor transmission is a key factor in addressing residual malaria. Outdoor latrines are characterized as semi-sheltered structures with humid environments, which may provide an ideal resting site for *Anopheles* species to feed on human blood and subsequently rest. This study aimed to quantify the abundance of adult mosquitoes in houses and outdoor latrines, as well as explore the environmental factors associated with mosquito abundance.

**Methods:**

Monthly mosquito sampling was conducted in 50 houses and their corresponding outdoor latrines using Prokopack aspirators from July 2023 to April 2024. Household interviews were conducted concurrently to collect data on the number of individuals sleeping in the houses and the quantity of bednets used within the households. In addition, blood meal sources were identified through polymerase chain reaction (PCR) analysis of blood-fed mosquitoes collected from December 2023 to April 2024.

**Results:**

Among anopheline species, the *An. funestus* group was the most common, followed by *An. gambiae* s.l. in both houses and latrines. In anophelines, the human blood index was 50.0% (*n* = 15) in houses and 33.3% (*n* = 4) in latrines, while bovine blood was 60.0% (*n* = 18) and 66.7% (*n* = 8), respectively. Ventilated improved pit (VIP) latrines were associated with a 61% decrease in *An. gambiae* s.l. abundance (adjusted incidence rate ratio [aIRR] = 0.39, 95% confidence interval [CI] = 0.16–0.96) and a 62% decrease in *Culex quinquefasciatus* abundance (aIRR = 0.38, 95% CI = 0.24–0.60), compared with pit latrines. The presence of a bathing space in latrines was associated with a 23% increase in *Cx. quinquefasciatus* abundance (aIRR = 1.88, 95% CI = 1.23–2.89) compared with latrines without a bathing space. There was an inverse association between the number of individuals using the latrines and *Cx. quinquefasciatus* abundance (aIRR = 0.93, 95% CI = 0.89–0.97).

**Conclusions:**

VIP latrines were associated with a lower abundance of *An. gambiae* s.l. and *Cx. quinquefasciatus* compared with pit latrines, whereas the presence of a bathing space in latrines was associated with a higher abundance of *Cx. quinquefasciatus* compared with latrines without bathing spaces. Integrative public health programs that promote the construction of VIP latrines as a low-cost intervention may provide co-benefits for both sanitation and vector control.

**Supplementary Information:**

The online version contains supplementary material available at 10.1186/s13071-025-07011-7.

## Background

Vector control measures, such as indoor residual spraying (IRS) and long-lasting insecticidal nets (LLINs), are effective in reducing malaria transmission by decreasing *Anopheles* populations and lessening vector–human contact [1–3]. IRS reached 88.4% of the targeted population in malaria-endemic countries in 2023 [4], and the percentage of households owning at least one LLIN in sub-Saharan Africa increased from 5% in 2000 to 73% in 2023 [4].

In Kenya, IRS campaigns targeting house structures was conducted using pyrethroid-based insecticides from 2005 to 2012 [3, 5]. These campaigns were interrupted owing to the emergence and spread of pyrethroid resistance in anopheline malaria vectors [6, 7]. Since 2017, non-pyrethroid IRS campaigns have been implemented in Migori and Homa Bay Counties, malaria-endemic regions located along Lake Victoria [8, 9]. IRS coverage in the target areas has consistently exceeded 90% [9, 10]. In parallel, free mass LLIN distribution campaigns in Kenya were conducted in 2006, initially targeting children under 5 years of age. Since 2012, bednets have been distributed every 3 years to achieve universal LLIN coverage, with one net for every two people [10–13]. LLINs are also provided for all pregnant women at their first antenatal clinic visit and for children under 1 year of age when they first attend a vaccination clinic [13]. The percentage of households owning at least one LLIN increased to 54.2% in 2022 from 5.9% in 2003 [14, 15].

Despite these concerted efforts for malaria vector control, malaria remains a leading cause of mortality in sub-Saharan Africa, including Kenya [9, 10, 16]. While the implementation of IRS and LLINs has reduced indoor *Anopheles* densities [17, 18], prolonged exposure to pyrethroids used in IRS and LLINs has led to the emergence of insecticide resistance among *Anopheles* species [19–23], shifts in vector species composition [24–26], and changes in feeding and resting behaviors [26–30]. For instance, in western Kenya, vector control has specifically reduced indoor densities of anthropophagic *An. gambiae* sensu stricto (s.s.) and the *An. funestus* group [17, 18], yet *An. arabiensis* persists even after IRS implementation owing to its zoophagic behaviors [7]. In addition, anophelines have altered their feeding patterns by shifting activity to evening and later morning hours, when people are not under bednets [28, 31–33].

Since sleeping under a net provides a physical barrier to prevent malaria transmission even in the presence of insecticide resistance [1, 2], transmission primarily occurs when individuals are not under nets, such as during outdoor activities. Reducing outdoor transmission is a key factor in addressing residual malaria, and some studies have been conducted to compare mosquito abundance and species composition between outdoor and indoor settings using various methods [28, 30–34]. Although most outdoor sampling has focused on areas around houses, few studies have sampled mosquitoes in outdoor latrines, where vector control measures are typically not implemented. These semi-sheltered structures with a humid environment [35] may provide an ideal location for *Anopheles* species [36] to feed on human blood and rest afterward. Consequently, latrine users may be exposed to *Anopheles* species in latrines between evening and morning, when the mosquitoes are active, potentially contributing to residual malaria transmission.

Most studies of mosquito-borne illnesses in western Kenya focus on *Anopheles* species and malaria; however, outbreaks of chikungunya fever, O’nyong-nyong, Rift Valley fever, and West Nile fever, transmitted by *Culex* and *Aedes* species, have also occurred in East Africa [37–39]. In addition, bancroftian filariasis has been detected from *Mansonia* species in Ghana [40]. Therefore, it is crucial to understand the behaviors of these mosquitoes alongside *Anopheles* species.

The present study aimed to identify the abundance, composition, and blood meal sources of adult mosquitoes in houses and outdoor latrines, and to explore whether environmental factors were associated with mosquito abundance. Understanding how various structures interact with mosquito feeding and resting behaviors can inform integrative prevention strategies that maximize efficient reductions in disease burden.

## Methods

### Study area

**Fig. 1:**
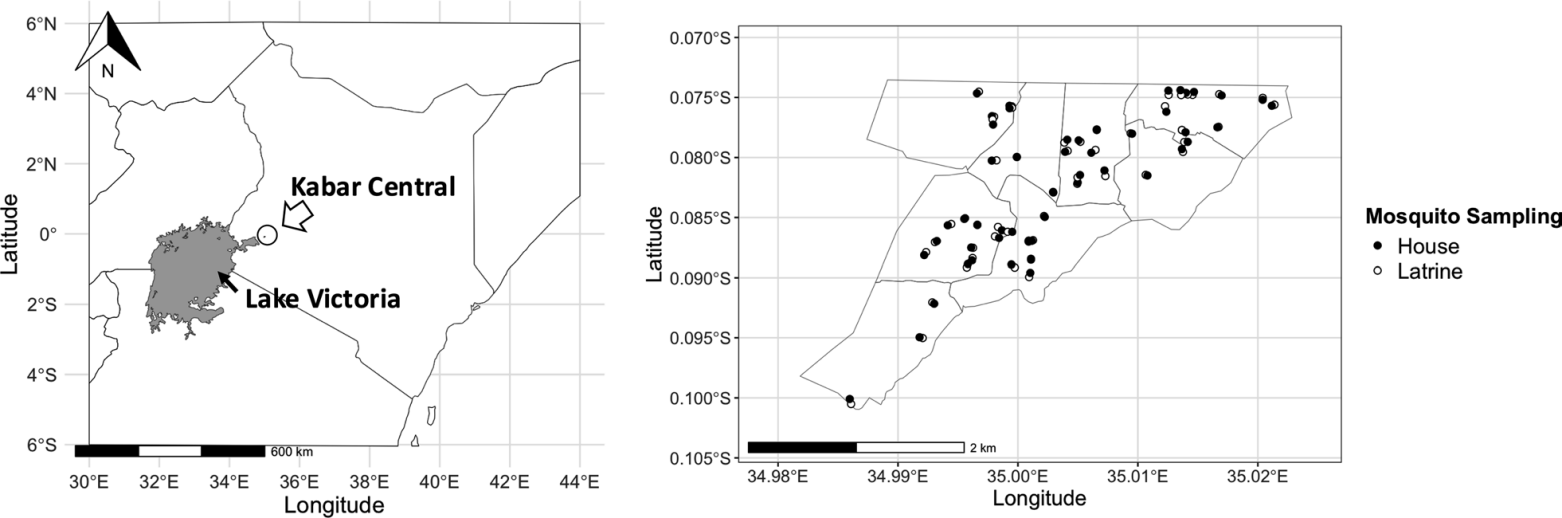
Study area and sampling locations. Maps showing Kenya and Kabar Central (study area) (left), and mosquito sampling houses and latrines (right). The Kenya map is based on data from Natural Earth (https://www.naturalearthdata.com/), and the Kabar Central map was provided by the author, based on self-collected Global Positioning System (GPS) data and Geographic Information System (GIS) data

The study area was defined as Kabar Central sublocation (6.2 km^2^) in western Kenya, located 30 km east of Kisumu (Fig. 1). It is approximately 1200 m above sea level. A total of 631 residential compounds were enumerated in the study area, comprising 1018 households with a total of 3898 residents. The rainfall pattern is bimodal, with a long rainy season from April to June and a short rainy season from November to December. Malaria occurs year-round with seasonal variations in transmission [41]. The primary vectors are *An. gambiae* sensu lato (s.l.), including *An. gambiae* s.s. and *An. arabiensis*, and the *An. funestus* group [9, 30]. The majority of the population are of Luo ethnicity and are subsistence farmers, while some are employed by local commercial sugarcane and rice growers. As the study area is not an IRS-targeted area, no IRS campaigns have been implemented there since they were interrupted in 2012, whereas LLINs were distributed in 2020 [42, 43].

In the study area, typical houses were constructed with mud walls, iron roofs, and open eaves. Outdoor latrines were either pit or ventilated improved pit (VIP) latrines, with iron or wooden doors; floors made of cement, tile, or mud; walls constructed from brick, iron, or mud; iron roofs; and open eaves. Pit latrines consist of a simple pit, and VIP latrines include a ventilation pipe to reduce odors and control flies. Regardless of whether the latrine is a pit or VIP type, some have an adjacent bathing space (Additional File 2: Supplementary Fig. S1). At night, some households place a bucket inside the house for individuals to use as an alternative to the outdoor latrine for collecting excreta.

### Data collection

#### Inclusion and exclusion criteria

Inclusion criteria: The present study was part of an investigation on latrine use and malaria transmission, which targeted individuals aged 4 years or older. Therefore, we included households that had at least one child aged 4–17 years, had a typical house (i.e., mud walls, iron roof, open eaves), and had a typical outdoor latrine (i.e., iron or wooden doors; cement, tile, or mud floors; brick, iron, or mud walls; a pit or VIP latrine; iron roof; open eaves). Exclusion criteria: Although closed eaves can prevent mosquito entry, only 4% (*n* = 44) of houses and 5% (*n* = 22) of latrines in the study areas had closed eaves. Therefore, houses and latrines with closed eaves were excluded to minimize the effects of structure on mosquito entry [44]. We also excluded houses with indoor latrines and those with multiple outdoor latrines to ensure comparability within the samples. The inclusion and exclusion criteria were applied to household data from the enumeration period in June 2023, as latrines in the study area are often constructed with low-durability materials and frequently require material replacements.

#### Mosquito sampling

Prior to data collection, we provided 2 days of training to four field personnel covering research ethics, basic entomology, and household survey administration. The participant houses were randomly selected from fully enumerated lists of Kabar Central residences that met the criteria. Monthly mosquito sampling was conducted from July 2023 to April 2024 in 50 houses and their corresponding outdoor latrines (including attached bathing spaces, if present) using Prokopack aspirators, which directly collect resting mosquitoes [45] (Additional File 2: Supplementary Fig. S1). Sampling was performed over a 10-month period between 6:00 a.m. and 8:30 a.m [46] over a 5-day period, with ten households sampled per day. If a household withdrew during the sampling period, a replacement was identified through another random draw from the enumerated list.

Mosquitoes sampled between July 2023 and February 2024 were stored in a freezer (−20 °C) at the field office. In March 2024, the chilled mosquitoes were transported to Homa Bay by car (approximately a 2-h drive) and stored in a freezer (−20 °C). Mosquitoes sampled between March and April 2024 were transported directly to Homa Bay.

#### Household survey

Interviews with household heads (adults) were conducted concurrently with mosquito sampling and entered into an electronic database, REDCap (Research Electronic Data Capture) [47]. We collected monthly data on the number of individuals sleeping in the houses and the quantity of bednets used within the households, along with mosquito sampling. The following environmental variables were collected once: cattle ownership (yes/no); presence of a bucket in the house; type of outdoor latrine (pit/VIP) (Additional File 2: Supplementary Fig. S1); presence of a bathing space in the latrine (yes/no); latrine door, floor, and wall materials; and the number of individuals potentially using the latrine (i.e., residents aged 4 years or older) [48].

#### Remote sensing data

Satellite imagery was obtained from the USGS EarthExplorer platform (https://earthexplorer.usgs.gov/). Normalized difference vegetation index (NDVI) for buffers around each sampling location was calculated monthly from July 2023 to April 2024. Imagery was extracted from bands 4 (red) and 5 (near-infrared radiation, NIR) from the Landsat 8–9 Operational Land Imager and Thermal Infrared Sensor (OLI/TIRS) C2 level 2 (30 m resolution), with 20–30% cloud cover. Monthly imagery was selected on the basis of proximity to mosquito sampling dates and minimal cloud cover [49]. The buffers used were 250 m, 500 m, 750 m, and 1000 m to account for the average mosquito flight distance [50, 51].

The NDVI ranges from −1 to +1, with values interpreted as follows: values of 0.1 or less, including negative values, represent barren rock, sand, or snow; values between approximately 0.2 and 0.5 correspond to shrubs and grasslands or senescing crops; and values between approximately 0.6 and 0.9 indicate temperate and tropical forests or crops. NDVI is a normalized transformation of the NIR to red reflectance ratio, as shown below [52, 53]:

$${\text{NDVI }} = \frac{{{\text{NIR}} - {\text{Red}}}}{{{\text{NIR}} + {\text{Red}}}}$$

Land surface temperatures (LST) were extracted for the same resolution and buffers mentioned above. LST was calculated using bands 10 from Landsat 8–9 OLI/TIRS C2 level 2 and converted to Celsius, as shown below [54]:

$$\begin{aligned} {\text{Temperature in Kelvin }} & = \, \left( {0.00341802 \, x{\text{ band }}10} \right) \, + \, 149.0 \\ {\text{Temperature in Celsius }} & = {\text{ Temperature in Kelvin }} - \, 273.15 \\ \end{aligned}$$

### Laboratory analysis

#### Species identification

Mosquitoes were grouped by genus using the keys of Gillies and Coetzee [55] and the Walter Reed Biosystematics Unit [56]. Female mosquitoes were further classified by their blood-feeding status (unfed, fed, half gravid, and gravid) under a dissecting microscope. Samples were stored at −20 °C until processing.

#### Blood meal sources

Blood meal sources were identified in 176 (30%) of female blood-fed mosquitoes collected from July 2023 to April 2024 (*n* = 578), owing to budget constraints. We focused on mosquitoes sampled between December 2023 and April 2024, as these more recent samples were considered less susceptible to the effects of unstable storage temperatures in the field office, compared with those collected in earlier months. The analyzed samples included 66 of 72 blood-fed mosquitoes collected in December 2023 and all blood-fed mosquitoes collected from January to April 2024 (*n* = 116).

DNA was extracted from abdominal blood using a simple Chelex protocol [57]. Cytochrome-B primers employed were Human 741F, Cow 121F, Goat 894F, Pig 573F, and Dog 368F, along with the universal primer UNREV1025 [58, 59]. PCR reactions were prepared using 2.0 µl of extracted DNA and DreamTaq Green PCR Master Mix (Thermo Fisher Scientific^™^ Waltham, MA, USA). The PCR conditions were as follows: initial denaturation of 95 °C for 3 min; 35 cycles of template denaturation at 95 °C for 30 s, primer annealing at 58 °C for 30 s, and amplicon extension at 72 °C for 1 min; and a final extension at 72 °C for 6 min. PCR products (5 µl) were separated on a 1.5% agarose gel alongside 10 µl of a 100 base pair (bp) DNA ladder for size comparison. Expected band sizes were 334 bp for human, 581 bp for cow, 132 bp for goat, 453 bp for pig, and 680 bp for dog.

### Data analyses

#### Characteristics of latrines

Characteristics of latrines and households were summarized. Proportions were computed for categorical variables. The mean and standard deviation (SD), and median and range were computed for numeric variables.

#### Mosquito sampling

The mosquito data are summarized as the total number of mosquitoes collected, the number of mosquitoes per house and per latrine, the proportion of blood-fed mosquitoes for each species, sex and blood meal status, and mosquito blood meal sources. For the proportion of blood-fed mosquitoes, the human blood index (HBI, proportion of blood meals from a human host [60]) and the bovine blood index (BBI, proportion of blood meals from a bovine host) were calculated on the basis of successfully amplified samples.

#### Comparison between houses and latrines

##### Mosquito abundance

We used generalized linear mixed models (GLMMs) to investigate the association between mosquito sampling locations (i.e., houses and outdoor latrines) and mosquito abundance, including anophelines and culicines. Since the mosquito count data were over-dispersed (the variances were larger than the means) and contained many zeros, we evaluated Poisson, negative binomial, zero-inflated Poisson, and zero-inflated negative binomial GLMMs. Model selection for each outcome was conducted on the basis of Bayesian information criterion (BIC). A negative binomial model with linear parameterization was selected for the outcome of the *An. funestus* group counts. Negative binomial models with quadratic parameterization were chosen for the outcomes of *An. gambiae* s.l. and *Cx. quinquefasciatus* counts. Monthly NVDI and LST were included as covariates in the models. The optimal buffer for each outcome was selected for using BIC. Moran’s I was then performed on the final models to assess the spatial autocorrelation. The same procedures were applied to all subsequent models.

##### Mosquito composition

Binomial proportional GLMMs were employed to assess the association of the sampling locations with mosquito composition. Three outcomes were modeled: the proportion of anophelines relative to total mosquitoes (anophelines and culicines), the proportion of *An. gambiae* s.l. relative to anophelines, and the proportion of the *An. funestus* group relative to anophelines. Monthly NVDI and LST were included as covariates in the models.

#### Factors influencing mosquito abundance in separate house and latrine datasets

We explored factors associated with mosquito abundance separately for house and latrine datasets. Poisson, negative binomial, zero-inflated Poisson, and zero-inflated negative binomial GLMMs were applied as described above and model selection was based on BIC. For houses, a negative binomial model with quadratic parameterization was selected for *An. gambiae* s.l. and *Cx. quinquefasciatus*, while a negative binomial model with linear parameterization was chosen for the *An. funestus* group. The predictors for houses included the number of people sleeping in a house, number of bednets used, having a bucket, owning cattle, NDVI, and LST. For outdoor latrines, a negative binomial model with linear parameterization was selected with *An. gambiae* s.l., a Hurdle Poisson model was chosen for the *An. funestus* group, and a negative binomial model with quadratic parameterization was selected for *Cx. quinquefasciatus*. The predictors for latrines included owning cattle, type of outdoor latrine, having a bathing space in the latrines, door material, the number of individuals potentially using the latrine, NDVI, and LST. A generalized variance inflation factor (GVIF) was used to assess collinearity among the covariates, and no covariates had a GVIF greater than three [61]. The GVIFs for floor and wall materials in latrines were between 2.7 and 2.6. Since these variables were related to the presence of a bathing space next to a latrine (i.e., latrines with a bathing space typically had cement/tile floor and brick/cement or iron walls), they were excluded from the dataset.

Since mosquito sampling was conducted repeatedly in houses and outdoor latrines, sampling locations and months were treated as random effects. NDVI and LST were standardized owing to their narrow range, ensuring better numerical stability and comparability in the analyses. We assumed that missing values were at random, and they were excluded from all analyses. All tests were two-sided, and the significance level was set at 0.05. ArcGIS Pro 3.3 (ESRI Corporation, Redlands, CA, USA) was used to process the polygon map of the study area, and R (version 4.0.2) was used for all analyses [62].

### Ethical statement

The study received ethical approval from the Kenya Medical Research Institute Scientific and Ethics Review Unit (KEMRI-SERU) (ref no. SERU4736) and the University of Arizona Institute Review Board, with the University of Arizona deferring primary oversight to KEMRI. Prior to the study, the field personnel visited households to explain the study’s purpose, procedures, potential risks and benefits, and voluntary participation of participating in the study. Written informed consent was obtained from all household heads (adults).

## Results

**Fig. 2:**
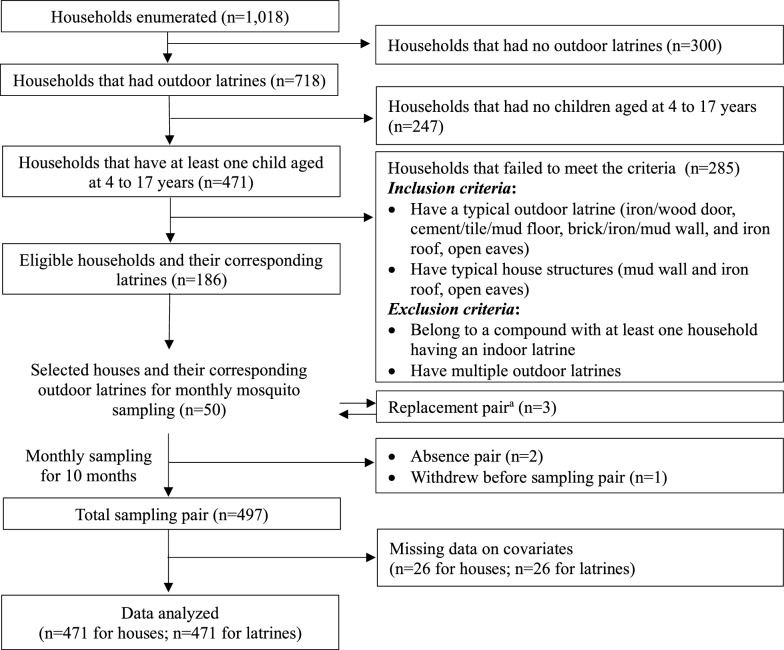
Flowchart showing selection of households for mosquito sampling from July 2023 to April 2024. ^a^ Three pairs were replaced with new ones in the same manner when a household withdrew during the sampling period. One withdrew before sampling, and the others withdrew after sampling. *VIP* ventilated improved pit

Of the 1018 households enumerated, 300 households had no outdoor latrines, 247 had no children aged 4–17 years, and 285 did not meet the structural criteria. Of the 186 households eligible for the present study, 50 households were randomly selected (Fig. 2). Although mosquitoes were sampled in houses and latrines from the 50 households over 10 months, sampling was conducted in 47 households in August and 49 households in October owing to the absence of the owners. Three pairs of houses and latrines were replaced with new ones. Missing data on bednet use and household characteristics occurred in 26 observations, resulting in the removal of three households and their corresponding latrines. Two latrines were updated during the sampling period. In total, 471 observations from 52 outdoor latrines were used for data analyses (Fig. 2).

Of 52 outdoor latrines, pit latrines (*n* = 37, 71.2%) were the most common, followed by VIP latrines (*n* = 37, 28.8%) (Table 1 and Additional File 2: Supplementary Fig. S1). About one third (*n* = 16, 30.8%) of latrines had a bathing space, and 44.0% (*n* = 22) of houses had cattle. The mean number of people sleeping in each house was 3.2 (SD = 1.7), and the mean number of bednets used per household was 1.8 (SD = 0.9). Three latrines no longer had doors between the enumeration and sampling phases (Table 1).

**Table 1:** Characteristics of households and latrines

Characteristics^a^	*n* (%)
Household	
No. of people sleeping in a house	
Mean (SD)	3.2 (1.7)
Median [range]	3 [0–11]
No. of bednets used	
Mean (SD)	1.8 (0.9)
Median [range]	0 [0–5]
Bucket^b^ in a house	
No	44 (88.0)
Yes	6 (12.0)
Presence of cattle	
No	28 (56.0)
Yes	22 (44.0)
Latrine	
Type	
VIP	15 (28.8)
Pit	37 (71.2)
Door	
Iron	41 (78.8)
Wood	5 (9.6)
Polythene/rag	3 (5.8)
No door	3 (5.8)
Floor	
Cement/tile	29 (55.8)
Mud	22 (42.3)
Wood	1 (1.9)
Wall	
Brick/cement	15 (28.8)
Iron	23 (44.2)
Mud	14 (26.9)
Have a bathing space in a latrine	
No	36 (69.2)
Yes	16 (30.8)
Potential number of people using a latrine^c^	
Mean (SD)	6.9 (4.8)
Median [range]	6 [1–26]

^a^Three households were replaced with new ones during the sampling periods. Although mosquitoes were sampled from a total of 53 households over 10 months, data on households and latrines were missing for 3 households. Data from two latrines were updated because they were reconstructed during the sampling period. Therefore, the total number of latrines was 52, while the total number of data other than latrines was 50

^b^At night, some households place a bucket inside of the house for potential use by individuals to collect excreta as an alternative to using an outdoor latrine

^c^Number of residents aged 4 years or older

*n* number, *SD* standard deviation, *VIP* ventilated improved pit

### Species abundance and composition

Of the 361 female anophelines collected in houses, the *An. funestus* group (*n* = 217, 60.1%) was the most common followed by *An. gambiae* s.l. (*n* = 140, 38.8%), *An. coustani* (*n* = 3, 0.8%), and *An. pharoensis* (*n* = 1, 0.3%). Similarly, among the 155 female anophelines found in latrines, the *An. funestus* group (*n* = 96, 61.9%) was the most common followed by *An. gambiae* s.l. (*n* = 52, 33.5%) and *An. coustani* (*n* = 7, 4.5%) (Additional File 1: Supplementary Table S1). The abundance of female *Cx. quinquefasciatus* (766 in houses; 696 in latrines) was more than double that of female anophelines in both houses and latrines. Of the 471 sampling events conducted at each of the house and latrine locations, the proportion of events in which at least one mosquito of each species was caught was as follows: 13.0% (*n* = 61) for houses and 7.1% (*n* = 33) for latrines for *An. gambiae* s.l., and 19.6% (*n* = 92) for houses and 9.1% (*n* = 43) for latrines for the *An. funestus* group.

### Seasonality

**Fig. 3:**
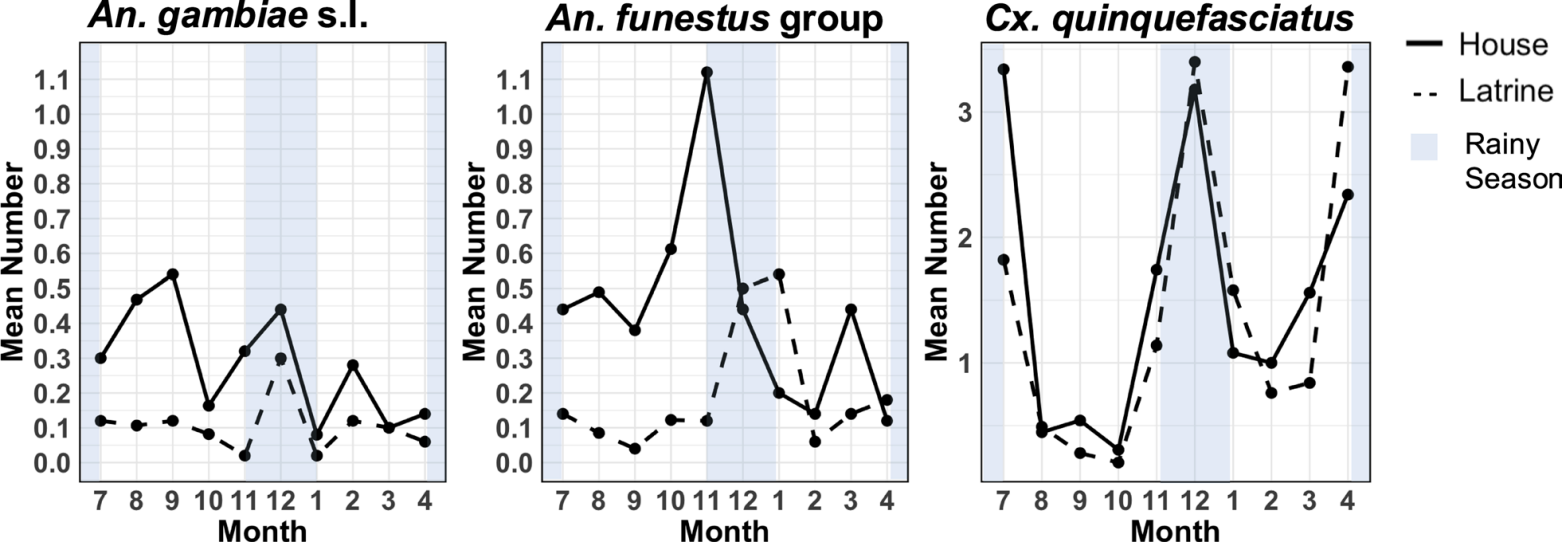
Mean number of *An. gambiae* s.l., *An. funestus* group, and *Cx. quinquefasciatus* sampled monthly from houses and latrines between July 2023 and April 2024. Mosquitoes were sampled in houses (solid line) and outdoor latrines (dashed line). The mean of mosquitoes over the 10-month sampling periods in houses was 0.28 (SD = 1.2) for *An. gambiae* s.l., 0.44 (SD = 1.3) for *An. funestus* group, and 1.56 (SD = 4.1) for *Cx. quinquefasciatus*. The mean in latrines was 0.10 (SD = 0.5) for *An. gambiae* s.l., 0.19 (SD = 1.0) for *An. funestus* group, and 1.40 (SD = 4.9) for *Cx. quinquefasciatus*. *An* *Anopheles*, *Cx* *Culex*, *s.l.* sensu lato

The abundance of *An. gambiae* s.l. in houses and latrines showed overall similar trends except in August and September, when the number increased in houses while remaining stable in latrines (Fig. 3). The abundance of *An. gambiae* s.l. in houses and latrines increased in December during the short rainy season (November–December). The peak number of the *An. funestus* group occurred in November for houses and in December and January for latrines. The mean number of *Cx. quinquefasciatus* in houses and latrines exceeded two in July (post-long rainy season), December (short rainy season), and April (long rainy season, April–June) (Fig. 3). The mean number of mosquitoes over the 10-month sampling period in houses was 0.28 (SD = 1.2) for *An. gambiae* s.l., 0.44 (SD = 1.3) for the *An. funestus* group, and 1.56 (SD = 4.1) for *Cx. quinquefasciatus*. The mean in latrines was 0.10 (SD = 0.5) for *An. gambiae* s.l., 0.19 (SD = 1.0) for the *An. funestus* group, and 1.40 (SD = 4.9) for *Cx. quinquefasciatus*.

### Sex and blood meal status

Overall, male mosquitoes comprised half or more of the *An. gambiae* s.l., *An. funestus* group, and *Cx. quinquefasciatus* (Table 2). The proportion of blood-fed *An. gambiae* s.l. was 62.9% (*n* = 88) in houses and 42.3% (*n* = 22) in latrines, while the proportion of blood-fed *An. funestus* group was 65.9% (*n* = 143) in houses and 16.7% (*n* = 16) in latrines (Table 2). A few *Ma. Africana*, *Ma. uniformi*, and *Ae. aegypti* were also collected in both houses and latrines. While blood-fed *Mansonia* species were found in houses, collected *An. coustani* and *Aedes aegypti* consisted only of unfed females in both houses and latrines (Table 2).

**Table 2:** Sex and blood meal status of mosquitoes collected in houses and latrines

	*An. gambiae* s.l., *n* (%)	*An. funestus* group, *n* (%)	*An. coustani*, *n* (%)	*An. pharoensis*, *n* (%)	*Ma. africana*, *n* (%)	*Ma. uniformis*, *n* (%)	*Ae. aegypti*, *n* (%)	*Cx. quinquefasciatus*, *n* (%)
House								
Gravid	22 (15.7)	27 (12.4)	0	1 (100)	0	0	0	153 (19.7)
Half gravid	1 (0.7)	4 (1.8)	0	0	0	0	0	30 (3.9)
Fed	88 (62.9)	143 (65.9)	0	0	3 (60.0)	3 (60.0)	0	207 (26.7)
Unfed	29 (20.7)	43 (19.8)	3 (100)	0	2 (40.0)	2 (40.0)	5 (100)	385 (49.7)
Male	136	266	0	0	0	0	0	0
Latrine								
Gravid	12 (23.1)	17 (17.7)	0	0	1 (33.3)	2 (66.7)	0	132 (19.1)
Half gravid	0	1 (1.0)	0	0	0	0	0	5 (0.7)
Fed	22 (42.3)	16 (16.7)	0	0	0	0	0	96 (13.9)
Unfed	18 (34.6)	62 (64.6)	7 (100)	0	2 (66.7)	1 (33.3)	3 (100)	459 (66.3)
Male	88	223	2	0	0	0	5	0

The proportion was calculated among female mosquitoes (gravid, half gravid, fed, and unfed)

*Ae* *Aedes*, *An* *Anopheles*, *Cx* *Culex*, *Ma* *Mansonia*, *s.l.* sensu lato

### Blood meal sources

Of the mosquitoes analyzed for blood meal sources using PCR, approximately half did not yield amplifiable host DNA and were thus excluded from analysis (*n* = 49, 50.0% in houses; *n* = 26, 53.1% in latrines) (Additional File: Supplementary Table S2). The HBI was lower than the BBI in both houses and latrines among the successfully amplified samples. The HBI in anophelines was 50.0% (*n* = 15) in houses and 33.3% (*n* = 4) in latrines, while the BBI was 60.0% (*n* = 18) in houses and 66.7% (*n* = 8) in latrines. The HBI for *An. gambiae* s.l. was 46.2% (*n* = 6) in houses and 14.3% (*n* = 1) in latrines, while the HBI for the *An. funestus* group was 52.9% (*n* = 9) in houses and 60.0% (*n* = 3) in latrines. The HBI for *Cx. quinquefasciatus* was 64.7% in houses (*n* = 11) and 63.6% (*n* = 7) in latrine. *Ma. africana* contained a human blood meal (*n* = 1, 100%), while *Ma. uniformis* contained a bovine blood meal (*n* = 1, 100%) (Additional File 1: Supplementary Table S2).

### Mosquito abundance and composition between houses and latrines

Samples from latrines showed a 48% lower abundance of *An. gambiae* s.l. (adjusted incidence rate ratio [aIRR] = 0.52, 95% confidence interval [CI] = 0.29–0.94) and a 57% lower abundance of the *An. funestus* group (aIRR = 0.43, 95% CI = 0.29–0.64), compared with samples from houses. *Cx. quinquefasciatus* abundance was not statistically different between latrines and houses (aIRR = 0.80, 95% CI = 0.59–1.10) (Table 3).

**Table 3:** The association of female mosquitoes in latrines with abundance and species composition in houses as a reference

Outcome	IRR/OR^a,b^	aIRR/aOR^a,b^
	(95% CI)	(95% CI)
Abundance^c^		
*An. gambiae* s.l	0.49^*^ (0.26–0.92)	0.52^*^ (0.29–0.94)
*An. funestus* group	0.42^*^ (0.26–0.69)	0.43^*^ (0.29–0.64)
*Cx. quinquefasciatus*	0.80 (0.58–1.12)	0.80 (0.59–1.10)
Subfamily or species composition		
Anophelines divided by anophelines and culicine	0.72^*^ (0.52–0.99)	0.73^*^ (0.53–0.99)
*An. gambiae* s.l. divided by anophelines	1.02 (0.63–1.65)	0.95 (0.60–1.50)
*An. funestus* group divided by anophelines	1.02 (0.72–1.46)	1.01 (0.72–1.41)

^a^IRR and aIRR were used for abundance outcomes, while odds ratios (ORs) and adjusted (a)ORs were used for subfamily or species composition

^b^Adjusted for standardized NDVI and LST

^c^A negative binomial model with linear parameterization was selected for the outcome of *An. funestus* group. Negative binomial models with quadratic parameterization were selected for the others

^*^Statistically significant

Households and sampling months were treated as random factors. Anophelines included *An. gambiae* s.l., *An. funestus* group, *An. coustani*, and *An. pharoensis*. Culicine included *Ma. africana*, *Ma. uniformis*, *Cx. quinquefasciatus*, and *Ae. aegypti*

*a* adjusted, *Ae* *Aedes*, *An* *Anopheles*, *CI* confidence interval, *Cx* *Culex*, *IRR* incidence rate ratios, *LST* land surface temperature, *Ma* *Mansonia*, *NDVI* normalized difference vegetation index, *OR* odds ratio, *s.l.* sensu lato

The odds of anophelines relative to culicines in latrines were 27% lower than in houses (adjusted odds ratio [aOR] = 0.73, 95% CI = 0.53–0.99). No significant differences were observed between latrines and houses in the odds of *An. gambiae* s.l. relative to other anophelines (aOR = 0.95, 95% CI = 0.60–1.50) or in the odds of the *An. funestus* group relative to other anophelines (aOR = 1.01, 95% CI = 0.72–1.41) (Table 3). No spatial autocorrelation was observed in the analyses (*P* > 0.05).

### Factors associated with mosquito abundance in houses

Unadjusted and adjusted models showed similar trends. Only NDVI was associated with the abundance of *An. gambiae* s.l., *An. funestus* group, and *Cx. quinquefasciatus* in houses. A one-unit increase in standardized NDVI was associated with a 52% decrease in *An. gambiae* s.l. abundance (aIRR = 0.48, 95% CI = 0.33–0.70), a 50% decrease in the *An. funestus* group abundance (aIRR = 0.50, 95% CI = 0.39–0.64), and a 23% decrease in *Cx. quinquefasciatus* abundance (aIRR = 0.77, 95% CI = 0.62–0.95) (Table 4). The optimal buffers used for NDVI and LST were 750 m for *An. gambiae* s.l. and 1000 m for the *An. funestus* group and *Cx. quinquefasciatus*. Both buffers showed the same mean value for NDVI (mean = 0.29, SD = 0.01) and LST (mean = 35.2, SD = 2.5) (Additional File 1: Supplementary Table S3). No spatial autocorrelation was observed in the analyses (*P* > 0.05).

**Table 4:** Factors associated with mosquito abundance in houses (*n* = 471 observations)

Parameter	*An. gambiae* s.l.^a^		*An. funestus* group^b^		*Cx. quinquefasciatus*^a^	
	IRR (95% CI)	aIRR (95% CI)	IRR (95% CI)	aIRR (95% CI)	IRR (95% CI)	aIRR (95% CI)
No. of people sleeping in a house	0.74 (0.45–1.23)	0.85 (0.55–1.32)	0.82 (0.57–1.17)	0.92 (0.69–1.24)	1.11 (0.86–1.42)	1.13 (0.88–1.45)
No. of bednets used	0.97 (0.77–1.21)	0.97 (0.78–1.20)	0.97 (0.85–1.11)	0.96 (0.85–1.09)	1.07 (0.96–1.20)	1.05 (0.94–1.18)
Bucket in a house^c^						
No	1	1	1	1	1	1
Yes	0.43 (0.09–2.04)	0.37 (0.09–1.48)	0.95 (0.58–2.21)	0.88 (0.39–1.98)	1.21 (0.60–2.41)	1.29 (0.66–2.50)
Presence of cattle						
No	1	1	1	1	1	1
Yes	1.28 (0.52–3.16)	1.22 (0.58–2.59)	1.13 (0.58–2.21)	1.05 (0.63–1.73)	1.14 (0.73–1.78)	1.09 (0.72–1.67)
NDVI^d^ (standardized)	0.45^*^ (0.31–0.66)	0.48^*^ (0.33–0.70)	0.50^*^ (0.39–0.63)	0.50^*^ (0.39–0.64)	0.80^*^ (0.65–0.99)	0.77^*^ (0.62–0.95)
LST^d^ (standardized)	1.36 (0.92–2.01)	1.29 (0.90–1.87)	1.04 (0.74–1.46)	0.92 (0.67–1.27)	0.73 (0.50–1.06)	0.69 (0.47–1.01)

^a^Negative binomial with quadratic parameterization model

^b^Negative binomial with linear parameterization

^c^At night, some households place a bucket inside the house for potential use by individuals to collect excreta as an alternative to using an outdoor latrine

^d^ 750 m buffer for *An. gambiae* s.l.; and 1000 m buffer for *An. funestus* group and *Cx. Quinquefasciatus*

^*^Statistically significant

*a* adjusted, *An* *Anopheles*, *CI* confidence interval, *Cx* *Culex*, *IRR* incidence rate ratios, *LST* land surface temperature, *NDVI* normalized difference vegetation index, *s.l.* sensu lato

### Factors associated with mosquito abundance in latrines

Unadjusted and adjusted models showed similar trends. VIP latrines were associated with a 61% decrease in *An. gambiae* s.l. abundance (aIRR = 0.39, 95% CI = 0.16–0.96) and a 62% decrease in *Cx. quinquefasciatus* abundance (aIRR = 0.38, 95% CI = 0.24–0.60), compared with pit latrines. The presence of a bathing space in latrines was associated with an 88% increase in *Cx. quinquefasciatus* abundance (aIRR = 1.88, 95% CI = 1.23–2.89) compared with latrines with no bathing space. Each additional individual using the latrines was associated with a 7% decrease in *Cx. quinquefasciatus* abundance (aIRR = 0.93, 95% CI = 0.89–0.97). In addition, a one-unit increase in standardized NDVI was associated with a 65% decrease in the *An. funestus* group abundance (aIRR = 0.35, 95% CI = 0.19–0.63) and a 30% decrease in *Cx. quinquefasciatus* abundance (aIRR = 0.70, 95% CI = 0.58–0.84) (Table 5). The buffers used for NDVI and LST were 250 m for *Cx. quinquefasciatus*, 500 m buffer for the *An. funestus* group, and 750 m buffer for *An. gambiae* s.l. These buffers showed the same mean values for NDVI (mean = 0.29, SD = 0.01) and LST (mean = 35.2, SD = 2.5) (Additional File 1: Supplementary Table S3). No spatial autocorrelation was observed in the analyses (*P* < 0.05).

**Table 5:** Factors associated with mosquito abundance in latrines (*n* = 471 observations)

Parameter	*An. gambiae* s.l.^a^		*An. funestus* group^b^		*Cx. quinquefasciatus*^c^	
	IRR (95% CI)	aIRR (95% CI)	IRR (95% CI)	aIRR (95% CI)	IRR (95% CI)	aIRR (95% CI)
Presence of cattle						
No	1	1	1	1	1	1
Yes	1.14 (0.56–2.35)	1.08 (0.54–2.15)	1.50 (0.39–5.77)	0.99 (0.32–3.11)	1.22 (0.75–1.99)	1.17 (0.74–1.72)
Type of latrine						
Pit	1	1	1	1	1	1
VIP	0.63 (0.27, 1.45)	0.39^*^ (0.16–0.96)	0.57 (0.12–2.65)	1.14 (0.30–4.26)	0.53^*^ (0.32–0.88)	0.38^*^ (0.24–0.60)
Have a bathing space in the latrine						
No	1	1	1	1	1	1
Yes	1.24 (0.59–2.60)	1.70 (0.77–3.77)	0.57 (0.12–2.65)	1.07 (0.31–3.71)	1.26 (0.74–2.14)	1.88^*^ (1.23–2.89)
Latrine door						
No door/rag	1	1	1	1	1	1
Iron or wood	1.04 (0.29–3.71)	1.43 (0.40–5.11)	1.44 (0.18–11.70)	1.00 (0.17–5.87)	1.69 (0.72–3.98)	1.63 (0.84–3.16)
No. of individuals potentially using the latrine	0.94 (0.85–1.03)	0.91 (0.83–1.01)	0.98 (0.84–1.15)	0.90 (0.77–1.05)	0.99 (0.94–1.04)	0.93^*^ (0.89–0.97)
NDVI^d^ (standardized)	1.35 (0.92–1.99)	1.27 (0.88–1.85)	0.38 (0.22–0.68)	0.35^*^ (0.19–0.63)	0.71^*^ (0.57–0.88)	0.70^*^ (0.58–0.84)
LST^d^ (standardized)	1.24 (0.90–1.71)	1.29 (0.92–1.80)	1.11 (0.83–1.50)	1.11 (0.85–1.46)	1.12 (0.73–1.73)	1.06 (0.72–1.57)

Households and sampling month were treated as random factors

^a^Negative binomial with linear parameterization

^b^Hurdle Poisson model

^c^Negative binomial with quadratic parameterization model

^d^250 m buffer for *Cx. quinquefasciatus*; 500 m buffer for *An. funestus* group; 750 m buffer for *An. gambiae* s.l

^*^Statistically significant

a adjusted, *An* *Anopheles*, *CI* confidence interval, *Cx* *Culex*, *IRR* incidence rate ratios, *NDVI* normalized difference vegetation index, *LST* land surface temperature, *s.l.* sensu lato, *VIP* ventilated improved pit

## Discussion

Our study showed that, among anopheline species, the *An. funestus* group was the most commonly found, followed by *An. gambiae* s.l. in houses and latrines. *An. gambiae* s.l. and the *An. funestus* group were less abundant in latrines compared with houses. The proportion of anophelines among mosquitoes collected in latrines was lower than in houses. Higher NDVI, i.e., increasing vegetation around the houses, was associated with a decrease in the abundance of *Anopheles* and *Culex* species in houses. In latrines, VIP latrines were associated with a reduction in the abundance of *An. gambiae* s.l. and *Cx. quinquefasciatus*. However, the presence of a bathing area in latrines was associated with an increased abundance of *Cx. quinquefasciatus*.

In the present study, the *An. funestus* group was dominant in both houses and latrines. High LLIN coverage has been shown to reduce the abundance of *An. gambiae* s.s. owing to its susceptibility to pyrethroids in LLINs [17, 18, 25, 29]. Therefore, *An. gambiae* s.l., which may include the sibling species *An. gambiae* s.s. and *An. arabiensis*, may have relatively decreased in abundance in our study area [7, 30]. *An. gambiae* s.s. is primarily anthropophilic (preferring human hosts) and endophilic (biting and resting indoors) [63]. In contrast, *An. arabiensis* exhibits opportunistic behavior, being highly zoophagic (feeding on animals) and exophilic (resting outside houses), though it can also be anthropophagic and endophilic [63]. As *An. arabiensis* tended to be more prevalent than *An. gambiae* s.s. in outdoors environments, including pit shelters [64], the *An. gambiae* s.l. collected from latrines in the present study possibly included more *An. arabiensis* than *An. gambiae* s.s. [17, 18]. Although sample size was small, HBI was higher than BBI in latrines. Given that over half of the *An. funestus* group were unfed and this species has been reported to be active in the late morning, around 11 a.m. [7, 28], they may have been host-seeking around latrines. Individuals using latrines during that time may be at risk of exposure.

A very small number of *An. pharoensis* and *An. coustani* were collected in the present study, and both species are capable of transmitting malaria [26, 65–67]. Although all *An. coustani* collected were unfed females or males, continued monitoring is warranted, as *An. coustani* has been documented to have high entomological inoculation rates [65, 66, 68].

A few *Ma. africana* and *Ma. uniformi* were found in both houses and latrines and some fed on human blood. Although these species can be a vector for bancroftian filariasis [69–71], the parasite has not been observed in these species in Kenya yet [72]. *Cx. quinquefasciatus*, frequently found in houses and latrines, has been reported to have high levels (80–90%) of susceptibility to *Wuchereria bancrofti* in Kenya [73]. Furthermore, *Cx. quinquefasciatus* may have contributed to a chikungunya outbreak in Mombasa, Kenya [74]. Since *An. coustani*, *Ma. Africana*, *Ma. uniformi*, and *Cx. quinquefasciatus* are potential vectors of diseases such as malaria, lymphatic filariasis, and chikungunya, long-term mosquito surveillance, including pathogen testing, is needed to understand their dynamics.

No associations were observed between household characteristics and mosquito abundance. In previous studies, thatched roofs have been reported to increase the abundance of *Anopheles* species, while closed eaves have been shown to decrease their abundance [75–77]. However, our study focused on houses with iron roofs and open eaves, which were a common design feature in our study area, and did not include houses with thatched roofs or closed eaves. This restriction may have created more standardization among house structures, which could have resulted in similar mosquito abundance. Previous studies have demonstrated that higher densities of household members were associated with an increase in mosquito abundance due to the release of concentrated carbon dioxide [75, 78]. However, the high LLIN coverage in the present study area (96.9% of households with at least one bednet, unpublished) may have contributed to the relatively low mosquito abundance [17, 18], minimizing the impact of the number of people sleeping in houses.

In addition, our results showed that an increase in the NDVI was associated with a decrease in *Anopheles* and *Culex* species. Given the low standard deviation of NDVI, vegetation density at the sampling location showed little variability. These results suggest that even slight increases in vegetation density may contribute to reductions in mosquito abundance. *An. gambiae* s.l. larvae inhabit small, temporary sunlit pools, *An. funestus* larvae are found in large, semi-permanent water bodies with aquatic vegetation, and *Cx. quinquefasciatus* larvae are commonly found in stagnant water [79–81]. Given the habitat preferences of these species, the averaged NDVI within the buffer, and the fact that NDVI does not directly reflect the presence of water bodies, small pools that support *An. gambiae* s.l. and *Cx. quinquefasciatus* may form on bare ground (i.e., areas with lower NDVI), whereas semi-permanent water bodies, such as rivers, may also have relatively low surrounding vegetation compared with agricultural fields. As a result, mosquito abundance may be lower in areas with higher NDVI vales than in those with lower NDVI. Further long-term monitoring is necessary to better understand the association between NDVI and mosquito abundance.

A previous study showed that VIP latrines were associated with reduced diarrheal illness in children under 5 years of age compared with unimproved pit latrines [82]. In our study, VIP latrines were also associated with lower mosquito abundance than pit latrines. VIP latrines may help reduce humidity and odor through ventilation, creating a less hospitable environment for mosquitoes, as observed in houses with increased ventilation [77]. If research on a larger sample size indicates that this holds true, the added benefit of VIP design may further justify their construction and use in the community.

Although the number of individuals using latrines may contribute to mosquito abundance by increasing concentrated odors, the present study indicated that this factor was associated with decreased *Culex* species abundance. Mosquito-preferred environments may encourage caretakers to take preventive measures, such as increasing the frequency of cleaning and using ash to remove odor and dry the latrines [83]. In addition, the presence of a bathing area in the shared latrine structure may provide a humid environment, which was likely associated with an increase in *Cx. quinquefasciatus* abundance [84].

These findings highlight the potential benefits of VIP latrines in reducing mosquito abundance and raise concerns about bathing spaces adjacent to latrines as potential mosquito habitats. Further research is needed to clarify the mechanisms underlying the association between latrine type and mosquito abundance, particularly in relation to odor and moisture. Nonetheless, maintaining well-ventilated, improved latrines as a low-cost intervention may provide co-benefit for both sanitation and vector control by reducing mosquito–human contact. These insights can inform vector control strategies that incorporate latrine design and maintenance in peri-domestic environments in malaria-endemic regions.

### Limitations

First, species identification and sporozoite detection for *An. gambiae* s.l. and the *An. funestus* group, and pathogen detection for *Mansonia* and *Culex* species were not conducted owing to resource constraints. Further studies are needed to better understand residual malaria transmission and other mosquito-borne infections by detecting pathogens in mosquitoes. Second, over half of the blood meal sources were not identified owing to non-amplified host DNA. Although we did not attempt re-extractions or repeat PCRs owing to resource constrains, one possible reason for the DNA degradation is unstable storage temperatures. Another possibility is that the mosquitoes had acquired the blood earlier, allowing sufficient time for the midgut digestive enzymes to degrade the DNA [85]. Given the small samples and potential bias, further study is needed to better understand blood meal sources. Third, the frequency and duration of nighttime latrine use, and bathing were not recorded in the present study. These behaviors may influence mosquito abundance in latrines. Passive monitoring of latrine use with a passive infrared motion detector may be useful for measuring the frequency of use at night [86]. Fourth, our study focused on typical houses and latrines, and on households with at least one child within the study areas. Therefore, our findings may not be generalizable to settings where other types of structures, such as latrines with closed eaves, or households without children, are common. Fifth, some mosquitoes may have exited the structures through non-door entrances or open eaves before sampling, potentially leading to an underestimation of mosquito abundance. The use of exit traps could improve the sampling outcome [87, 88]. Finally, mosquito sampling was limited by the small number of mosquitoes collected and the absence of year-round sampling. The small sample size may result in insufficient statistical power. A larger sample size with longitudinal sampling across multiple years would be needed to understand seasonal variations more comprehensively.

## Conclusions

VIP latrines were associated with a lower abundance of *An. gambiae* s.l. and *Cx. quinquefasciatus* compared with pit latrines, whereas the presence of a bathing space in latrines was associated with a higher abundance of *Cx. quinquefasciatus* compared with latrines without bathing spaces. Our findings provide novel evidence that outdoor latrines, particularly pit latrines and those with bathing spaces, serve as important locations associated with increased abundance of *An. gambiae* s.l. and *Cx. quinquefasciatus*. Integrative public health programs that promote the construction of VIP latrines as a low-cost intervention may provide co-benefits for both sanitation and vector control by reducing mosquito–human contact. Latrines and bathing areas should be considered in vector surveillance and control planning, as they may represent potential entomological risk factors for *Anopheles*- and *Culex*-borne diseases.

## Supplementary Information

Additional file 1.

Additional file 2 (Fig. S1. Type of latrines and mosquito sampling with a Prokopack aspirator. Pit latrine (A); ventilated improved pit (B); mosquito sampling in a latrine with a bathing space using a Prokopack aspirator (C).)

## Data Availability

Data supporting the main conclusions of this study are included in the manuscript.

## Abbreviations

Ae: Aedes
An.: Anopheles
BBI: Bovine blood index
BIC: Bayesian information criterion
Cx: Culex
DNA: Deoxyribonucleic acid
GIS: Geographic information system
GLMM: Generalized linear mixed model
GVIF: Generalized variance inflation factor
HBI: Human blood index
IRR: Incidence rate ratios
IRS: Indoor residual spraying
KEMRI: Kenya Medical Research Institute
LLIN: Long-lasting insecticidal nets
LST: Land surface temperature
Ma.: Mansonia
NDVI: Normalized difference vegetation index
NIR: Near-infrared
OLI/TIRS: Operational Land Imager and Thermal Infrared Sensor
OR: Odds ratio
PCR: Polymerase chain reaction
REDCap: Research Electronic Data Capture
s.s.: Sensu stricto
s.l.: Sensu lato
SD: Standard deviation
SERU: Scientific and Ethics Review Unit
USGS: United States Geological Survey
VIP: Ventilated improved pit
WASH: Water, sanitation, and hygiene
