# Supplementary material for: Structural features of outdoor latrines influence the abundance of Anopheles gambiae s.l. and Culex quinquefasciatus in a village in Kisumu County, western Kenya
Source: Parasit Vectors. 2025 Aug 27;18:364. doi: 10.1186/s13071-025-07011-7 (PMC12392610; doi:10.1186/s13071-025-07011-7)
Supplement: Supplementary file 1 — Additional file 1. [file 13071_2025_7011_MOESM1_ESM.docx]

| **Additional file 1: Table S1 Female mosquitoes sampled from July 2023 to April 2024** | | | | |
| --- | --- | --- | --- | --- |
|  | House | | Latrine | |
|  | n (%) | per night, median  [range] | n (%) | per night, median [range] |
| ***Anopheles*** |  |  |  |  |
| *An. gambiae* s.l. | 140 (38.8) | 0 [0, 15] | 52 (33.5) | 0 [0,6] |
| *An. funestus* group | 217 (60.1) | 0 [0, 14] | 96 (61.9) | 0 [0, 15] |
| *An. pharoensis* | 1 (0.3) | 0 [0,1] | 0 | 0 [0, 0] |
| *An. coustani* | 3 (0.8) | 0 [0, 2] | 7 (4.5) | 0 [0, 5] |
| ***Total*** | 361 (100) | 0 [0, 22] | 155 (100) | 0 [0, 26] |
| ***Mansonia*** |  |  |  |  |
| *Ma. africana* | 5 (50.0) | 0 [0, 1] | 3 (50.0) | 0 [0,3] |
| *Ma. uniformis* | 5 (50.0) | 0 [0,1] | 3 (50.0) | 0 [0, 2] |
| ***Total*** | 10 (100) | 0 [0,2] | 6 (100) | 0 [0,5] |
| *Ae. aegypti* | 5 | 0 [0, 2] | 3 | 0 [0,1] |
| *Cx. quinquefasciatus* | 776 | 0 [0, 73] | 696 | 0 [0, 97] |
| *Ae,* Aedes; An, *Anopheles*; *Cx*, *Culex*; *Ma*, *Mansonia*; n, number; s.l., sensu lato | | | | |
| Note: Of the 471 sampling events conducted for each of house and latrine, the proportion of the households in presence of mosquitoes: *An. gambiae s.l.* (13.0 % for house, 7.1 % for latrine)*, funestus group* (19.6 % for house, 9.1 % for latrine); *An. pharoensis* (0.2 % for house, 0% for latrine); *An. coustani* (0.4% for house, 0.6 % for latrine). | | | | |

| **Additional file 1: Table S2 Blood meal sources of mosquitoes collected from houses and latrines between December 2023 and April 2024.** | | | | | |
| --- | --- | --- | --- | --- | --- |
|  | *An. gambiae* s.l., n | *An. funestus group*, n | *Ma. Africana*, n | *Ma. uniformis*, n | *Cx. quinquefasciatus*, n |
| ***House*** |  |  |  |  |  |
| Human | 5 | 7 | 1 | 0 | 10 |
| Human/bovine | 1 | 2 | 0 | 0 | 0 |
| Human/dog | 0 | 0 | 0 | 0 | 1 |
| Bovine | 7 | 8 | 0 | 1 | 4 |
| Dog | 0 | 0 | 0 | 0 | 1 |
| Pig | 0 | 0 | 0 | 0 | 1 |
| No amplification | 13 | 8 | 0 | 1 | 27 |
| Not tested^a^ | 3 | 12 | 1 | 0 | 13 |
| ***Latrine*** |  |  |  |  |  |
| Human | 0 | 3 | 0 | 0 | 6 |
| Human/bovine | 1 | 0 | 0 | 0 | 0 |
| Human/dog | 0 | 0 | 0 | 0 | 1 |
| Bovine | 6 | 1 | 0 | 0 | 3 |
| Dog | 0 | 1 | 0 | 0 | 1 |
| No amplification | 5 | 4 | 0 | 0 | 17 |
| Not tested^a^ | 0 | 2 | 0 | 0 | 10 |
| *An*, *Anopheles*; *Cx*, *Culex*; s.l., sensu lato | | | | | |
| ^a^ Due to budget constraints, the identification was performed on a portion of the blood-fed mosquitoes collected in December 2023 and all blood-fed mosquitoes between January and April 2024 | | | | | |

| **Additional file 1: Table S3 Buffer, NDVI, and LST used for each outcome** | | | |
| --- | --- | --- | --- |
| Outcome | Buffer,  m | NDVI,  Mean (SD) | LST, ℃,  Mean (SD) |
| ***Abundance***^3^ |  |  |  |
| *An. gambiae* s.l. | 500 | 0.3 (0.01) | 35.2 (2.5) |
| *An. funestus* group | 1,000 | 0.3 (0.01) | 35.2 (2.5) |
| *Cx. quinquefasciatus* | 1,000 | 0.3 (0.01) | 35.2 (2.5) |
| ***Subfamily or species composition*** |  |  |  |
| Anophelines divided by anophelines and culicine | 1,000 | 0.3 (0.01) | 35.2 (2.5) |
| *An. gambiae* s.l. divided by anophelines | 250 | 0.3 (0.01) | 35.2 (2.5) |
| *An. funestus* group divided by anophelines | 250 | 0.3 (0.01) | 35.2 (2.5) |
| ***House*** |  |  |  |
| *An. gambiae* s.l. | 750 | 0.3 (0.01) | 35.2 (2.5) |
| *An. funestus* group | 1,000 | 0.3 (0.01) | 35.2 (2.5) |
| *Cx. quinquefasciatus* | 1,000 | 0.3 (0.01) | 35.2 (2.5) |
| ***Latrine*** |  |  |  |
| *An. gambiae* s.l. | 750 | 0.3 (0.01) | 35.2 (2.5) |
| *An. funestus* group | 500 | 0.3 (0.01) | 35.2 (2.5) |
| *Cx. quinquefasciatus* | 250 | 0.3 (0.01) | 35.2 (2.5) |
| *Ae*, *Aedes*; *An*, *Anopheles*; s.l., sensu lato; *Cx*, *Culex*; LST, land surface temperature; NDVI, normalized difference vegetation index; s.l., sensu lato | | | |
