# Supplementary figures and images for: Structural features of outdoor latrines influence the abundance of Anopheles gambiae s.l. and Culex quinquefasciatus in a village in Kisumu County, western Kenya
Source: Parasit Vectors. 2025 Aug 27;18:364. doi: 10.1186/s13071-025-07011-7 (PMC12392610; doi:10.1186/s13071-025-07011-7)

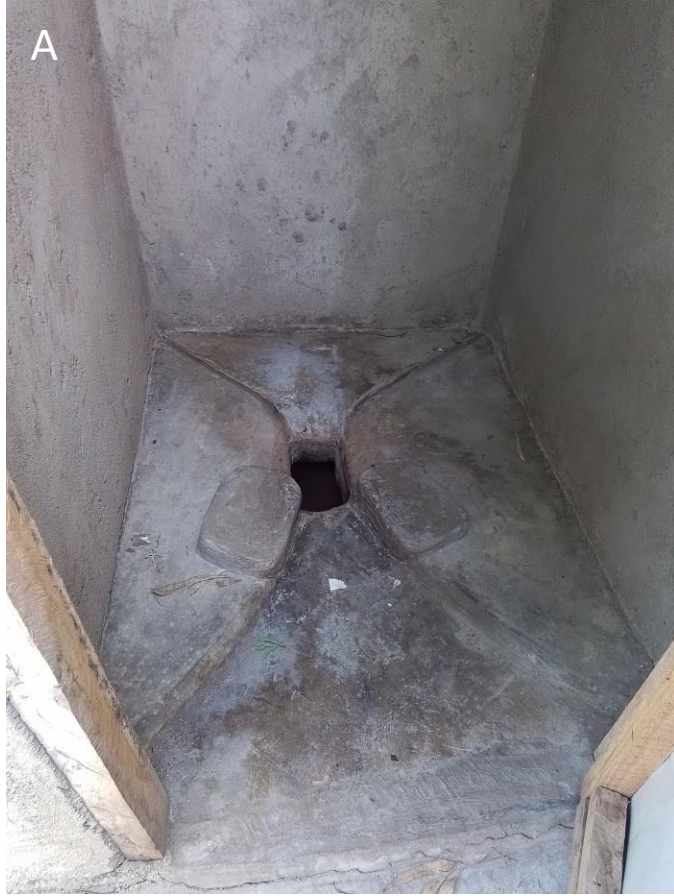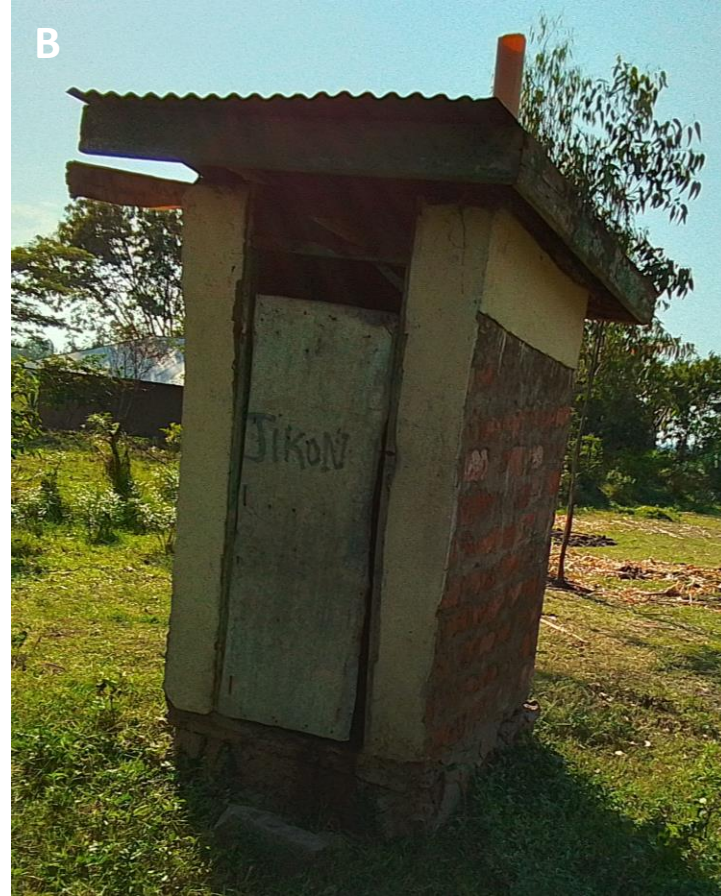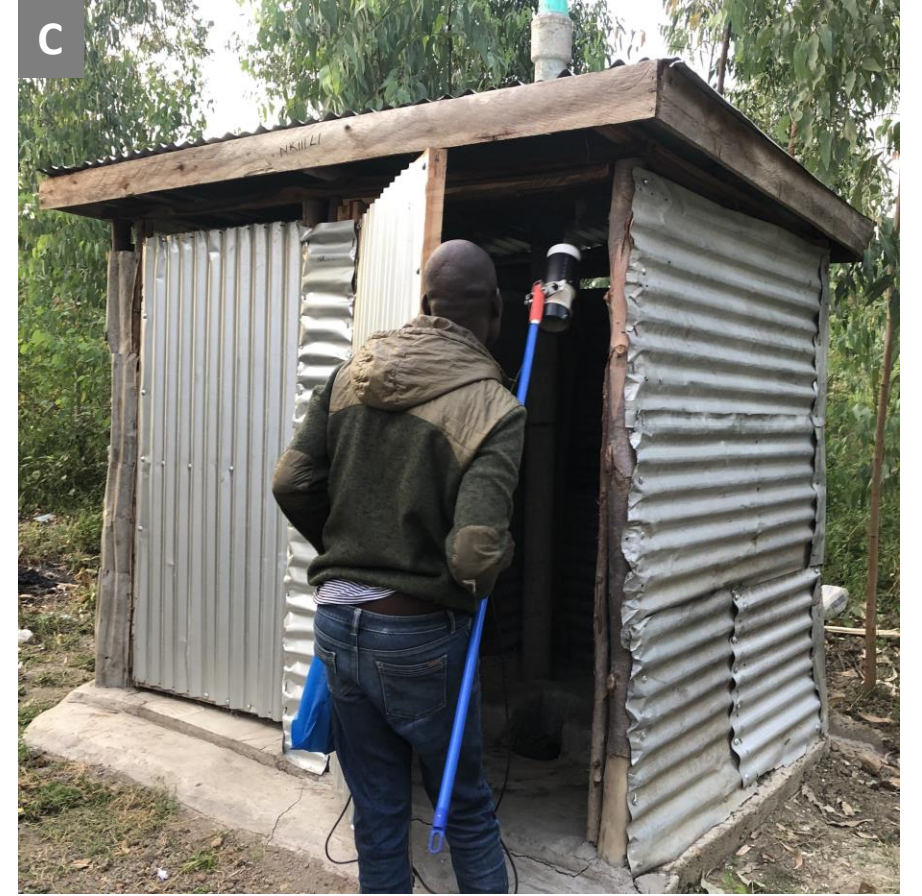

Supplement: Supplementary file 2 — Additional file 2 (Fig. S1. Type of latrines and mosquito sampling with a Prokopack aspirator. Pit latrine (A); ventilated improved pit (B); mosquito sampling in a latrine with a bathing space using a Prokopack aspirator (C).) [file 13071_2025_7011_MOESM2_ESM.pdf]
